# Feasibility of a Neural Network-Based Virtual Sensor for Vehicle Unsprung Mass Relative Velocity Estimation

**DOI:** 10.3390/s21217139

**Published:** 2021-10-27

**Authors:** Eldar Šabanovič, Paulius Kojis, Šarūnas Šukevičius, Barys Shyrokau, Valentin Ivanov, Miguel Dhaens, Viktor Skrickij

**Affiliations:** 1 Transport and Logistics Competence Centre, Transport Engineering Faculty, Vilnius Gediminas Technical University, 10223 Vilnius, Lithuania; paulius.kojis@vilniustech.lt (P.K.); viktor.skrickij@vilniustech.lt (V.S.); 2 Department of Mobile Machinery and Railway Transport, Transport Engineering Faculty, Vilnius Gediminas Technical University, 08101 Vilnius, Lithuania; sarunas.sukevicius@vilniustech.lt; 3 Department of Cognitive Robotics, Delft University of Technology, 2628 CD Delft, The Netherlands; b.shyrokau@tudelft.nl; 4 Automotive Engineering Group, Technische Universität Ilmenau, 98693 Ilmenau, Germany; valentin.ivanov@tu-ilmenau.de; 5 Tenneco Automotive Europe, 3800 Sint-Truiden, Belgium; MDhaens@Tenneco.com

**Keywords:** virtual sensor, automotive control, active suspension, vehicle state estimation, neural networks, deep learning, long-short term memory, sequence regression

## Abstract

With the automotive industry moving towards automated driving, sensing is increasingly important in enabling technology. The virtual sensors allow data fusion from various vehicle sensors and provide a prediction for measurement that is hard or too expensive to measure in another way or in the case of demand on continuous detection. In this paper, virtual sensing is discussed for the case of vehicle suspension control, where information about the relative velocity of the unsprung mass for each vehicle corner is required. The corresponding goal can be identified as a regression task with multi-input sequence input. The hypothesis is that the state-of-art method of Bidirectional Long–Short Term Memory (BiLSTM) can solve it. In this paper, a virtual sensor has been proposed and developed by training a neural network model. The simulations have been performed using an experimentally validated full vehicle model in IPG Carmaker. Simulations provided the reference data which were used for Neural Network (NN) training. The extensive dataset covering 26 scenarios has been used to obtain training, validation and testing data. The Bayesian Search was used to select the best neural network structure using root mean square error as a metric. The best network is made of 167 BiLSTM, 256 fully connected hidden units and 4 output units. Error histograms and spectral analysis of the predicted signal compared to the reference signal are presented. The results demonstrate the good applicability of neural network-based virtual sensors to estimate vehicle unsprung mass relative velocity.

## 1. Introduction

Nowadays, the automotive industry focuses intensely on Automated Driving (AD) as a promising solution to improve safety and comfort. The main functional components related to AD include perception, decision-making and vehicle control. New generation vehicles will need more information to accomplish these three tasks and ensure safe driving without human involvement. The required data can be gained using standard and novel sensors, Vehicle to Everything (V2X) communication and sensor fusion.

Sensors are crucial components needed for AD, as they provide the data required to perceive the environment and vehicle state estimation [1]. The first group includes laser imaging detection and ranging (LIDAR), radio detection and ranging (Radar) [2], ultrasonic distance sensor and a camera. These sensors are placed outside the vehicle, and measurement accuracy may be affected if covered by dirt, snow or ice. Cameras can be installed inside the cabin, which may prevent the disadvantages mentioned above. They can be applied to detect and track traffic objects, vision-based localisation and navigation, and capture textures and colours. Usage of stereo and infrared cameras can expand their uses for geometric parameter estimation, capturing objects in dark lighting and more [3,4,5]. The other group of sensors used for vehicle state estimation involves a global navigation satellite system (GNSS), inertial measurement unit (IMU), accelerometers, displacement sensors and wheel encoders. These sensors gather data about geolocation, vehicle position, angular rates and body’s accelerations. In addition, some novel sensors for wheel load reconstruction have been recently proposed [6,7]. To preprocess and filter the data, sensor fusion of multiple sensors’ data provides more reliable and accurate input data. For example, combining GNSS and IMU provides vehicle information about global position and velocity. Camera and LIDAR or Radar fusion provides 3D environment representation [1,2].

With an increasing number of measured, estimated and received data, virtual sensing emerged. The virtual sensor (VS) is a pure software sensor that estimates signals by combining and aggregating signals that it receives from physical or other VSs [8,9]. The VS is an abstraction for various types of underlying algorithms, including but not limited to Kalman filter estimators and Artificial intelligence-based regression and prediction algorithms. Virtual sensors have broad use cases. They are very useful in deriving physical quantities that cannot be measured directly [10,11], e.g., indexes of performance and efficiency. VS can also be used to replace or provide redundancy for cases where the installation of a physical sensor is challenging or sensors are unreliable or expensive. For example, pressure measurement in a shock absorber or force measurement in wheel carrier/bearing [12,13]. VS technology requires additional development costs but reduces repetitive maintenance costs [10]. At the same time, a decrease in the system’s physical parts increases the overall reliability. Diagnostic applications could be incorporated by observing and predicting the system’s state in advance or detecting machine degradation [14,15,16,17]. In the case of synthetic data use for VS, preparing a mathematical model requires high competencies and skills. A mismatch between the model and the actual system leads to failure and high inaccuracy. When experimental data are used for VS training, computing structures on which sensors operate require accurate measurements and large datasets that cover as many real-life cases as possible, including the rare ones. An insignificant error can generate a significant drift in the estimated signals [10,18].

VS are classified according to their development approach [10,14]: (*i*) measurement characteristics-based, (*ii*) modelling methods-based and (*iii*) application purpose-based. Measurement characteristics-based VSs are used to represent the system’s steady-state or transient measurements. Steady-state modelling is defined by the instant response to input variables and moderate changes in measured values compared to the system’s dynamics. A transient state type reacts slower due to complexity but allows a faster rate of change in input values. Modelling methods-based VS can be divided into data-driven, model-based or rule-based, considering modelling methods. Data-driven VSs are derived from historical data gathered by physical sensors. Emerging artificial intelligence methods such as neural networks have made breakthroughs in this type of VS. Model-based sensors operate using fundamental physical laws and mathematical relations between variables, which are the main part for equations used for Kalman filter estimators and other similar adaptive filtering approaches that use known models for estimation. Lastly, the rule-based VS utilises both approaches and relies on physical parameters and empirical models.

Virtual sensing is intensively used for automotive applications, for example, for passenger thermal comfort, the tire pressure monitoring system, powertrain applications, sprung mass state estimation [19,20] and others. VS may be a key technology of advanced control algorithms, enabling customisation of vehicle–human interaction.

The vehicle–human interaction by AD needs to be carefully designed and evaluated, taking into account different aspects. This requires revising ride comfort and safety of the vehicles in urban and countryside conditions as part of the task. The suspension design has a crucial influence on the ride comfort and handling of the vehicle [21]. The tuning of the passive suspension has limited applicability to improve the vehicle’s ride quality, as comfort and handling are conflicting objectives [22,23]. As a solution, semi-active or active suspension controlled by specialised control algorithms should be used.

During the last few decades, many strategies for suspension control have been developed. The majority of them use the velocity of sprung mass and velocity of unsprung masses as input parameters. In commercial systems, velocities are evaluated using integrated data from an IMU sensor placed on vehicle sprung mass and integrated/differentiated data from an accelerometer and displacement sensor placed on the vehicle unsprung mass. The accelerometer and displacement sensor combination is often used for unsprung mass velocity estimation. There is a lot of noise in the measurement data, and it is not easy to obtain acceptable results using only one sensor. The development of virtual sensors may solve the need for physical sensors on unsprung mass. Only a few works in this field have appeared in recent years.

One of the first works was Milanese et al. [24]. The authors investigated the problem of designing suitable linear virtual sensors focusing on the estimation of the relative vertical position and velocity between chassis and wheel, using the data from the accelerometer placed on the chassis or wheel. For the task, the Direct Virtual Sensor design technique has been proposed. In 2014, Pletschen and Badur [25] presented a new nonlinear suspension state estimation approach based on Kalman Filter theory and Takagi-Sugeno modelling. Wang et al. [26] proposed the Adaptive Kalman Filter to accurately estimate a vehicle’s suspension system state under different road conditions. Jeong et al. [27] proposed a strategy for relative suspension velocity estimation. The method consists of mathematical modelling and direct measurements provided by an IMU sensor. Vertical front suspension forces are calculated using governing equations combined with heave, roll and pitch accelerations measured by the sensor. Out of these forces, relative suspension velocity is derived. Vazquez et al. [28] provided a suspension state, road profile and transfer load estimation methodology using deflection sensors, accelerometers and gyrometers. The observation scheme used is a linear Kalman filter. Despite the approach’s simplicity, its robustness against uncertainty is remarkable.

As it can be seen, the most commonly used techniques for suspension state estimation involve a Kalman Filter; we propose a new approach in this investigation. Investigation of the feasibility of a Neural Network (NN) model-based virtual sensor for an unsprung mass relative velocity estimation is provided in this paper. NNs are increasingly used for sequence regression tasks, including mechanical state prediction [29] and lateral vehicle velocity estimation [30,31]. This leads to the assumption that similar methods can be used for virtual sensor development to estimate unsprung mass relative velocity. Based on [30], the Bidirectional Long–Short Term Memory (BiLSTM)-based NN model may provide a better performance compared to two layers of Long–Short Time Memory (LSTM) and 1-dimensional convolutional NN (1D CNN). Therefore, BiLSTM was selected for the NN-model of a proposed virtual sensor for unsprung mass relative velocity estimation in the presented research. The main advantage of such a data-driven VS is that the model for a physical process that connects input sensors with reference data is learned in a process called deep learning and requires no handcrafted equations that relate all input signals to output signals. This allows consideration of many more input sensors that were not considered earlier because no model included them. Neural network-based models can also provide robustness in relation to input data by reducing dependence on a single sensor. BiLSTM, as much as LSTM, are recurrent neural networks that are able to consider the last output, new input and state of the neuron to estimate output. They can consider a number of past samples and, if needed, may compensate for computation overhead by making estimations in advance. In addition, so far, during the research review, no research on VS based on the NN model for vehicle unsprung mass relative velocity estimation was found.

The main contributions of the research presented in this article are as follows. A data-driven VS for real-time application for vehicle unsprung mass relative velocity estimation using multiple sensor data of selected sample window size was developed in this research. Additionally, the proposed NN model-based virtual sensor was validated and tested on simulated data, with error data analysis, including time and frequency domains. In addition, error distribution was analysed using an error histogram. The model has a low enough computational overhead to provide output signal estimation for input data that were sampled at 100 Hz. Designed VS eliminates the need for physical sensors on vehicle unsprung masses. The output can be used for semi-active/active suspension control. The contributions of this article prove the feasibility of neural network-based virtual sensors for vehicle unsprung mass relative velocity estimation and provide a base for further research and implementation of VS for unsprung mass velocity estimation in the field of the automotive industry. The further structure of the paper is as follows. In Section 2, we present (i) a vehicle model, (ii) manoeuvres used for dataset creation and (iii) the BiLSTM-based Deep NN model and its optimisation algorithm. Section 3 provides the results of vehicle model validation. Hyperparameter optimisation and BiLSTM-based DNN structure selection are also provided in this section. Finally, we analyse virtual sensor validation and testing results using the Root Mean Squared Error (RMSE) of unsprung mass relative velocity and compare the accuracy of developed VS with results achieved by other researchers. Additionally, in Section 4, the discussion is performed, main conclusions are presented and further steps for system development are presented.

## 2. Materials and Methods

This section describes materials and methods used for data acquisition and development, validation and testing of the virtual sensor for vehicle relative velocity of unsprung mass. First, the vehicle mathematical model is presented, including software descriptions and scenarios. Second, an NN-model used for the virtual sensor is described, including the model structure and hyperparameter optimisation method.

### 2.1. Virtual Sensor for Vehicle Unsprung Mass Relative Velocity Estimation

**Figure 1:**
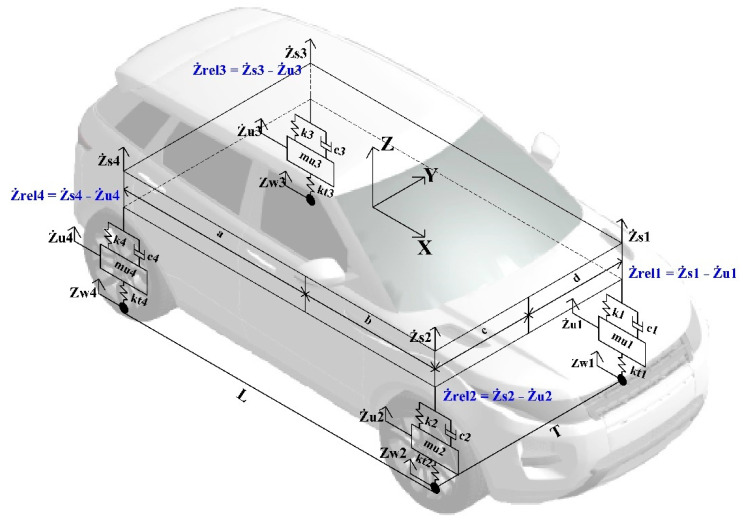
SUV dynamic model.

For vehicles equipped with semi-active or active suspensions, the velocities of sprung and unsprung masses are required to implement a control strategy. Sprung mass velocity can be measured using an IMU sensor placed on the sprung mass. The velocity of unsprung mass is commonly evaluated using data from accelerometer and displacement sensors. Our approach for VS creation was supervised learning with selected sensors input and recorded relative unsprung mass velocity data (Figure 1). In the picture, Ż_rel1,2,3,4_ are the relative velocities of the unsprung masses in the vertical directions for the front left (FL), front right (FR), rear left (RL) and rear right (RR) wheels; Ż_u1,2,3,4_ refer to the unsprung masses; Ż_s,1,2,3,4_ indicate sprung mass velocities; Z_w1,2,3,4_ are road roughness.

The vehicle model of a Sport Utility Vehicle (SUV) was built in the IPG CarMaker simulation platform. The model has been parametrised and validated using field test data from the proving ground; vehicle parameters are presented in Table 1. It is a modified electric Range Rover Evoque vehicle with onboard electric motors. The tire parametrisation using experimental data was performed to simulate tire dynamics.

Simulation data included 14 parameters as inputs: sprung mass accelerations in 3 directions (x, y, z), angular rates around these three axes, longitudinal vehicle velocity, front wheels’ steering angle and overall vehicle steering angle from steering system, angular velocities of the wheels. The vertical velocities of four unsprung masses were used as an output.

**Figure 2:**
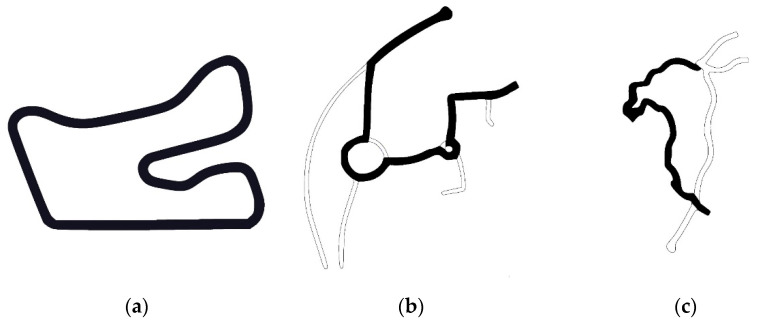
Selected tracks: (**a**) Hockenheimring; (**b**) Rural roundabouts road; (**c**) Heilbronn.

Test tracks and manoeuvres were selected with consideration of data collection for NN training and validation. More dynamic data must be collected for training; therefore, three scenarios were chosen from the standard scenarios in IPG CarMaker (Figure 2).

Hockenheimring track located in Germany has an overall length of 2.6 km. It is a one directional racing track with some straight sections and various corners. There all road can be used for manoeuvres such as hard cornering. Various speeds up to 120 km/h and driving manners were applied to cover most vehicle dynamics. Secondly, a two-line rural road roundabouts scenario was selected. This scenario contains braking and acceleration manoeuvres, lane changes and driving around the constant radius turns. The overall length driven by the vehicle on this track is 1.76 km, and the maximum speed was 75 km/h.

Furthermore, a model of Heilbronn road located in Germany was used. It has one lane for each direction. This curvy road contains inclinations and declinations. Additional user-defined stopping sections were used to induce acceleration and deceleration manoeuvres. The overall track length covered was 5.79 km with an average speed of 55 km/h. The driver model was modified to three different parameter sets featuring defensive, normal and aggressive driving styles in all mentioned test scenarios. Different longitudinal, lateral acceleration levels and varying cruising speeds define each driving style.

Data from Heilbronn and rural road tracks were used for training and Hockenheimring for validation. Additionally, constant radius cornering (ISO 4138:2012), obstacle avoidance, Sine with Dwell (ISO 19365:2016), bumpy road and slalom manoeuvres were simulated for NN dataset creation (see Section 3.3), and the data were used for NN testing.

### 2.2. BiLSTM-Based Deep Neural Network Model

To take advantage of sequential sensor data, a Bidirectional Long–Short Term Memory (BiLSTM)-based recurrent network was used. This neural network (NN) is attributed to recurrent neural networks (RNN) and can learn sequential data models and base predictions on past and current signal values. Model structure, the hyperparameter selection experiment and the results of validation and testing are presented in this section.

**Figure 3:**
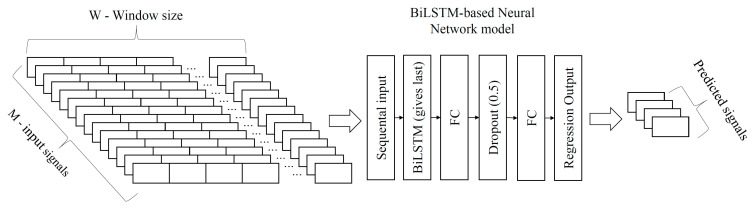
Structure of BiLSTM-based RNN, its inputs and outputs.

The structure of the selected RNN includes six layers; see Figure 3. NN model layers include sequence input layer, BiLSTM layer, one hidden fully connected (FC) layer, Dropout layer, one output FC layer and regression output. The sequential input layer lets in data from all input channels as a sample sequence of length defined by the window size parameter. This layer takes a sample count of M = 14 input signals. The count of samples equals selected window size W. These samples are fed into the BiLSTM layer. This layer includes extended LSTM units that propagate signals forward and backwards. This may improve model performance compared to LSTM. The BiLSTM layer consists of a memory cell and gates controlled by trainable neurons that learn when to forget, update and output the cell value considering cell memory value, last input value and current input value. BiLSTM output given for the last sample is taken as signal features extracted for a supplied sequence of input signals of window size W. This result is supplied to the hidden FC layer. This layer, together with the output FC layer, processes features extracted in the BiLSTM layer. The units count of the Output FC layer is equal to the outputs count of the virtual sensor. The dropout layer is between the hidden FC and the output FC layer. The dropout layer randomly zeros inputs of the next layer with defined probability. The proposed network used dropout with 0.5 probability. This reduces NN dependence on single features from BiLSTM as any future can be dropped, and at least two features are required to decide as the dropout probability is set to 0.5. Regression output provides estimations for the output signals.

The network’s input is sequential samples of all inputs M for window size W. Input data consists of 14 sensor data defined in Section 2.1. Table 2 provides input and output signal names and units that are used for the NN model. The input data samples are fed to the network sequentially, and only the output of BiLSTM for the last input sequence sample is forwarded to the next layer. Therefore, calculation duration in the BiLSTM layer depends linearly on window size W. As each BiLSTM unit performs the same calculations, matrix and vector calculation is used. As a result, calculation speed depends less on the overall unit count in NN layers such as BiLSTM and FC. All operations can be performed in one cycle of the parallel processing unit and especially graphical processing units (GPU). This network model used in the real-time situation would require holding input data samples that cover selected window size W. The model unit counts are limited to 512 and window size to 51. Therefore, model implementation on low-power devices and automotive onboard computers is feasible.

Even a tiny artificial NN has at least some hyperparameters. They are defined before training and are not optimised during the training process. However, hyperparameters have a significant impact on NN performance and need careful selection or optimisation. As hyperparameters are changed before training, NN training and validation operations are performed for each combination. Therefore, training itself is a long operation, and selecting hyperparameter takes longer. In order to formalise the process of hyperparameter selection, unique optimisation methods are used.

**Figure 4:**
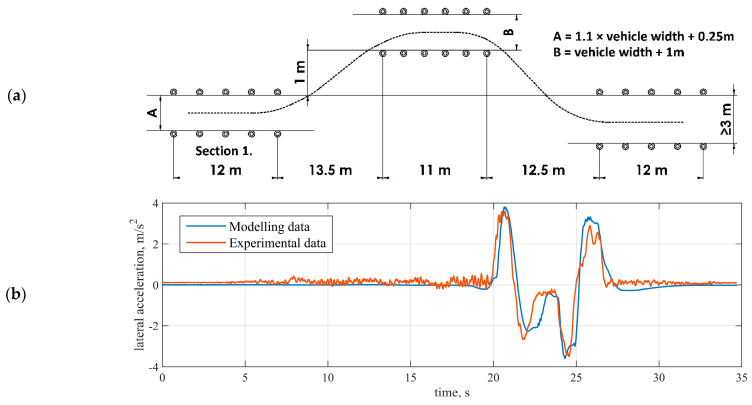
Obstacle avoidance test (ISO 3888-2:2011): (**a**) manoeuvre scheme; (**b**) sprung mass lateral accelerations during the test.

There are three main methods for hyperparameter optimisation: grid search, random search and Bayesian search. Grid search is the simplest but most computationally expensive. It involves iterating through the defined multi-dimensional grid of hyperparameters combinations. If the step size in this grid is small, the iterations count can become enormous, and this type of optimisation may take a very long time and many computation resources. It is possible that the grid is too sparse and will not provide the best possible solution. In addition, significant time will be spent on unpromising combinations. Random search has no defined grid, and parameter combinations are generated randomly; the random process may be faster compared to grid search if the probability distribution is uniform. Random search has shown that it can find good combinations faster compared to grid search. Bayesian optimisation is similar to random search. Instead of randomly selecting pairs, it analyses previous combination results, builds the Gaussian probability model and makes a new combination selection to improve the model (see Figure 4). It saves all combinations, always saving the best; it also provides an estimate that may not be tested yet but provides an even better performance. Hence, time is reduced and avoids the drawbacks of grid search.

## 3. Results

### 3.1. Vehicle Model Validation and Dataset Generation

The vehicle model was parametrised and validated on an IPG CarMaker-based simulation platform using field tests data from the proving ground [32]. The obstacle avoidance manoeuvre is presented in Figure 4 as a validation example, achieved accuracy RMSE = 0.39 m/s^2^. The test was performed to determine vehicle nature at a severe lane-change manoeuvre. Overall track length was 61 m. All sections were marked with cones of a minimum height of 500 mm. ISO 3888–2:2011 [33] does not specify a minimum or limit velocity level, but throttle application was stopped in 2 m after entering Section 1.

After vehicle model validation, the dataset was generated for NN training, validation and testing. All the roads and manoeuvres are described in Section 2, as well as input and output data.

### 3.2. Results of Hyperparameter Optimisation

The predefined window size for data samples and selectable unit counts in BiLSTM and FC layers were used during hyperparameter optimisation of the selected NN model. The window sizes of 3, 7, 11, 17, 19, 21, 25, 31 and 51 have been used. The bigger the window size, the more features can be extracted from the signals, especially the lower frequency and more complex features. On the other hand, a bigger window linearly increases computation duration to obtain the model’s final output of the sequence. Therefore, a trade-off between the duration of computation and accuracy should be introduced. The number of layers and their order in the network were selected manually, inspired by reviewed articles. First, we choose the smallest possible NN model to reduce computational overhead.

During the Bayesian Search, the unit counts in BiLSTM and FC layers are selected from range [1, 512]. The models with each selected combination of unit counts are trained and validated using MATLAB on a Nvidia Geforce 2080 Ti graphical processing unit (GPU). An adaptive moment estimation (ADAM) optimiser used for training with 0.001 learning rate and training was limited to 30 epochs. One epoch is one round of training on all available data, so 30 epochs mean 30 repetitions; each repetition lets us better choose the connection weights between artificial neurons inside the NN model. For each window size, the mini-batch size is selected to fill the GPU memory as much as possible, as models are pretty small and in cases of small window size do not utilise GPU completely. The total count of trained combinations was around 350.

**Figure 5:**
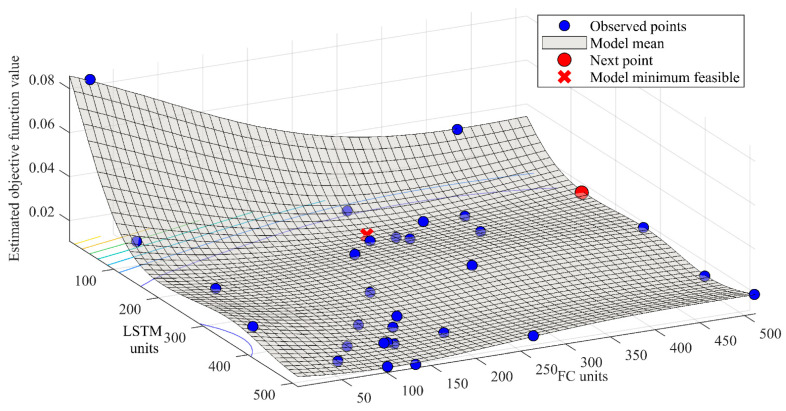
Bayesian search for NN model using sample window of 21.

RMSE of unsprung mass relative velocity was used as a metric for optimisation. As two parameters were optimised and one metric was used, a 3D mesh can be drawn to show the optimisation process. In Figure 5, hyperparameter optimisation mesh for a window size of 21 is shown.

Training and validation error graphs showed that RMSE reduces mainly in 15 epochs and not much after 25 epochs. There was no overfitting detected in graphs of the training process. The hyperparameter optimisation process wrapping the training would reject overfitting networks based on validation RMSE after each training.

In Figure 5, it can be observed that blue dots correspond to tested combinations, black for following possible combination, the red star shows the feasible model minimum point and red mesh shows the model mean. Under that mesh, there is a 2D graph with isolines of various colours. Blue points represent low values and yellow ones correspond to high values of the estimated objective, RMSE. This model helps the search algorithm to select the following points in hyperparameter space. However, the 2D graph of observed and estimated objective function value on the y axis and trial number on the x-axis is provided.

After a Bayesian search for 30–60 iterations for each window size, only the best network models were selected for each sample window size. The best models are presented with their hyperparameters, RMSE and relative calculation duration in Table 3. The calculation duration is based on sequential calculation (minibatch size of 1) of all validation samples, 19,800 minus (window size-1). As duration depends on processor speed, the presented calculation durations are considered only relatively, and calculation time is presented compared to the smallest sample window as a baseline. The calculation time measurement has been performed three times because of temporal dependencies on hardware performance. It is not possible to compare computation overhead to other methods reported in other articles because hardware, code and runtime environment optimisations differ and would not provide a reliable comparison. The current implementation of the proposed algorithm runs on Nvidia GTX 2080 Ti GPU. Real-time target machines are used for prototyping such algorithms in the automotive industry. Proposed VS may run considerably faster when running on the central processing unit (CPU), field programable gate array (FPGA) or digital signal processor (DSP) of a real-time target machine such as dSpace, because there will be no such latencies as are introduced by data transfer between CPU and GPU on personal computers.

**Figure 6:**
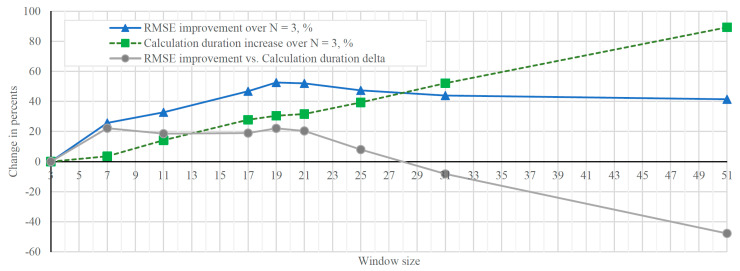
Relative performance and calculation time dependence on sample window size.

The results show that RMSE reduces when window sizes from 3–19 are used. Therefore, accuracy is being improved, and relative calculation time increases with sample window size growth. The graphs of relative performance and calculation duration are shown in Figure 6. This graph demonstrates that the size of the window of 19 brings the best improvement in performance compared to the increase in calculation duration; longer sample windows bring diminishing returns.

In Figure 6, the best RMSE improvement compared to windows size W = 3 is achieved using window size 19. RMSE improvement in percent and calculation duration delta shows that the most significant positive delta is achieved at a window size of 7 and 19. After evaluating these graphs, the conclusion can be made that the best sample window size is 19. This also brings the best compromise between accuracy and computation duration. Therefore, validation and testing results are provided for the window size 19 in the next section.

### 3.3. Virtual Sensor Validation and Testing

**Figure 7:**
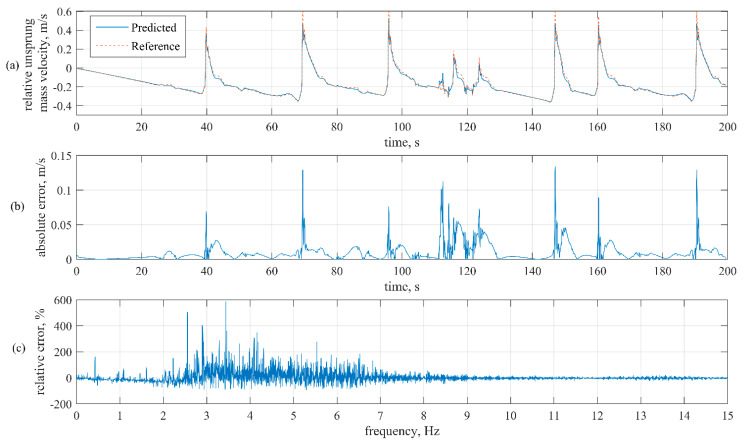
Comparison of predicted and reference signal: (**a**) time-domain graph of reference and predicted signal; (**b**) time-domain graphs of absolute error of predicted signal compared to reference; (**c**) relative error of frequency spectrum of a predicted signal.

This section provides and analyses virtual sensor outputs on validation and testing data compared to reference data, which are unsprung mass relative velocities provided as part of simulated vehicle parameters. The developed virtual sensor estimates unsprung mass relative velocity in the vertical direction. The time series and frequency diagrams of the original and predicted vertical velocity of the unsprung mass are presented in Figure 7.

The time-domain graphs of actual simulation output and NN model output for one wheel are provided for validation simulation of Hockenheimring with the normal driving scenario in Figure 7a.

In order to better present an actual difference between actual and estimated velocity, the corresponding absolute error is shown in Figure 7b. The max. absolute error is about 0.11 m/s while RMSE is 0.0081. The spikes in absolute error correspond with higher-frequency changes in unsprung mass relative velocity and acceleration.

**Figure 8:**
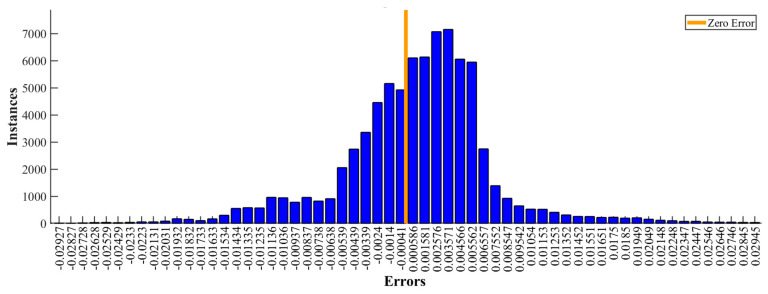
Error histogram for unsprung mass relative velocity prediction compared to the reference.

Frequency analysis has been performed to understand how the difference is spread over the spectrum of measurable frequencies. The spectrum relative error was calculated as the delta between predicted and reference signal spectrums divided by the spectrum of the reference signal. The results are shown in Figure 7c. Measurements are made at 100 samples per second; we provide graphs from 0–15 Hz based on primary and secondary ride quality assessment. The observed relative error is mostly between 0–10 Hz. The max. relative error is concentrated around 3 Hz. Additionally, in Figure 8, the error histogram shows how predicted signal error values are distributed.

In addition to Figure 7b, Figure 8 shows that most errors are between −0.01384 and 0.01402 m/s. A small disbalance around zero is related to the asymmetric bin ranges, considering that the form of distribution meets Gaussian distribution.

Complete testing procedure involved 3 validation and 20 testing simulations, and RMSE results calculated for each wheel (unsprung mass) relative velocity prediction separately (FL, FR, RL, RR) and overall RMSE of simulation. The results are shown in Table 4. Furthermore, the training RMSE was included to validate the training correctness, as RMSE on the training set should be smaller than on the validation and testing sets. There was not much noise in the higher frequency (Figure 7c). Therefore, the assumption is that augmenting input sensor data with simulated sensor and process noise may improve generalisation.

Besides the RMSE, Accuracy was calculated from normalised RMSE (NRMSE) as proposed in [25]:

(1) $$\mathrm{NRMSE}=\;\frac{\sqrt{\frac{1}{N}\sum_{n=1}^{N}{\left(\hat{{\dot{Z}}_{\mathrm{rel}}}-{\dot{Z}}_{\mathrm{rel}}\right)}^{2}}}{\sqrt{\frac{1}{N}\sum_{n=1}^{N}{\left({\dot{Z}}_{\mathrm{rel}}\right)}^{2}}},$$

(2) $$\mathrm{Accuracy}=100\;\cdot \;\left(1-\mathrm{NRMSE}\right),$$

where ${\dot{Z}}_{\mathrm{rel}}$—reference unsprung mass relative velocity on Z axis, $\hat{{\dot{Z}}_{\mathrm{rel}}}$—estimated relative unsprung mass relative velocity on Z axis, N—total samples in the tested scenario, *n*—current sample. Accuracy metric lets us compare the proposed method to other methods [25,26].

Based on Table 4, first, aggressive driving increases RMSE, which may be related to higher acceleration and jerk in vehicle motion, as unsprung mass movement depends on road profile and driver’s behaviour. Second, higher vehicle speeds increase the RMSE of prediction, as higher speed means higher frequencies, which can be explained by the fact that higher speed increases the frequency of change in unsprung mass relative velocity. Higher frequency signals caused by abrupt manoeuvres may include signal frequencies beyond Nyquist frequency for 100 Hz sampling rate, which may lead to aliasing in the spectrum.

In research performed by other researchers, authors use different vehicles, road types and manoeuvres. In [25], authors achieved an accuracy of 97.7% for relative vertical velocity; however, it is true only for one road type—rough country road and vehicle velocity of 50 km/h. In [26], the authors used absolute wheel velocity as the output. Their achieved results fall in the range from 30.6–95.9%. The best accuracy of the NN-based method proposed in this research is 99% for a constant turn with a radius of 60 m at 50 km/h test; it is the best result if we compare it with those available in the literature. Moreover, the average accuracy for all the tests is 91.2%. For bumpy roads, results are constantly low and vary from 74.3–77.9%. The worst results were achieved during a constant turn with a different radius at extremely high speeds. Similar road types may be added to the training dataset to increase accuracy; this will be solved in the future.

## 4. Discussion and Conclusions

This research aimed to develop a data-driven virtual sensor for unsprung mass relative velocity prediction based on other vehicle movement characterising sensor data, including IMU and steering-related sensors. The hypothesis was that this problem could be solved as a sequential signal regression task. A literature review showed that state-of-art multi-input sequence regression could be implemented using recursive NN, and BiLSTM-based models are currently achieving impressive results.

In order to implement an NN-based virtual sensor, training, validation and testing datasets are needed. These datasets were generated using an experimentally validated vehicle model, developed using software for advanced simulation of vehicle dynamics IPG Carmaker. The simulations covered two tracks and three various driving styles for training, one track and three different artificial driving styles for validation and five manoeuvres for testing with various parameters and road profiles.

The NN structure has been selected for multi-input sequential regression. The hyperparameter optimisation using Bayesian search was made to select the best parameters. The experiment evaluated the developed models’ performance as main metrics RMSE and relative calculation time were selected. Based on the simulations, the NN model with a window size of 19 provides the best performance improvement compared to the computation duration increase.

During the simulation studies, virtual sensor output signals were compared to the reference and analysed in the time and frequency domain. The best NN model demonstrates that the predicted signal is close to reference with an RMSE of 0.0081 and a maximum error of 0.11 m/s. Most errors are concentrated between −0.01384 and 0.01402 m/s, and the error distribution is Gaussian. Frequency domain analysis shows that most of the error is between 0–10 Hz, with peak values at 3 Hz.

The best accuracy of the NN-based method proposed in this research is 99%; it is the best result compared to the ones available in the literature. Moreover, the average accuracy for all the tests is 91.2%.

It can be concluded that VSs using an NN model such as BiLSTM are viable for unsprung mass relative velocity prediction. The final NN consists of 167 BiLSTM, 256 hidden FC units and 4 output FC units. This virtual sensor running on a computer with Nvidia Geforce 2080 Ti GPU can process one sample per 2.5 ms and provide up to a 400 Hz sample rate. The proposed algorithm implemented on a real-time target machine may provide even greater performance, which is part of future research. The ability of the NN model to provide prediction in advance may also be used to compensate for most computation overhead.

In the future, the development of a virtual sensor using a 1D Convolutional NN and comparison with the one developed in this research is planned. During the next steps, recorded experimental data from a car running on the proving ground will be used in addition to the simulation data that were used in this research. The sensitivity of VS on each input and robustness to sensor malfunction will be analysed. During the final stages of future research, the proposed virtual sensor will be assessed on a dSPACE real-time target machine during an on-road test. Additional future research may be required to account for any shortcomings prior to possible implementation in production vehicle systems.

## Figures and Tables

**Table 1:** Data used in mathematical model.

Parameter	Symbol	Value
Wheelbase	L	2.675 m
Distance between front axle and COG	b	1.439 m
Distance between rear axle and COG	a	1.236 m
Height of COG above ground	h	0.65 m
Vehicle mass	m	2442 kg
Total unsprung mass	m_u_	126.2 kg
Distance between left track and COG	d	0.778 m
Distance between right track and COG	c	0.847 m
Track width	T	1.625 m
Wheel rotational inertia	J	0.9 kg m^2^
Tire stiffness	K_t_	225,368 N/m
Loaded tire radius	R_l_	0.343 m
Tire size		235/55/R19
Pitch inertia		642.3 kg m^2^
Roll inertia		2892 kg m^2^
Yaw inertia		3231 kg m^2^

**Table 2:** Input and output sensor data used for training NN model.

Input Parameters			Output Parameters		
Input Nr.	Name	Units	Output Nr.	Name	Units
1	Sprung mass acceleration (X axis)	m/s^2^	1	Unsprung mass relative velocity (Z axis) front left	m/s
2	Sprung mass acceleration (Y axis)	m/s^2^	2	Unsprung mass relative velocity (Z axis) front right	m/s
3	Sprung mass acceleration (Z axis)	m/s^2^	3	Unsprung mass relative velocity (Z axis) rear left	m/s
4	Sprung mass angular rate (X axis)	deg/s	4	Unsprung mass relative velocity (Z axis) rear right	m/s
5	Sprung mass angular rate (Y axis)	deg/s			
6	Sprung mass angular rate (Z axis)	deg/s			
7	Vehicle’s longitudinal velocity	m/s			
8	Steering angle of front left wheel	deg			
9	Steering angle of front right wheel	deg			
10	Wheel speed of front left wheel	m/s			
11	Wheel speed of front right wheel	m/s			
12	Wheel speed of rear left wheel	m/s			
13	Wheel speed of rear right wheel	m/s			
14	Vehicle’s steering angle	deg			

**Table 3:** Hyperparameter optimisation results with performance and calculation duration comparison for each window size.

Window Size	Selected Parameters		RMSE	Relative Error, %	Calculation Duration, ms/Sample	Relative Calculation Duration, %
	BiLSTM Units	FC Units				
3	360	403	0.0171	100.0	1.89	100.0
7	502	295	0.0127	74.3	1.96	103.5
11	202	312	0.0115	67.3	2.16	114.2
17	512	111	0.0091	53.2	2.42	127.8
19	167	256	0.0081	47.4	2.47	130.5
21	137	298	0.0082	48.0	2.49	131.6
25	207	511	0.0090	52.6	2.63	139.4
31	116	345	0.0096	56.1	2.88	152.1
51	137	298	0.0100	58.5	3.58	189.3

**Table 4:** RMSE achieved in various training, validation and testing scenarios for each wheel and overall.

Scenario	RMSE					Accuracy, %
	FL	FR	RL	RR	Overall	
Heilbronn track, Aggressive driver (training)	0.0034	0.0033	0.0035	0.0034	0.0034	96.5
Heilbronn track, Offensive driver (training)	0.0021	0.0020	0.0021	0.0020	0.0021	97.5
Heilbronn track, Normal driver (training)	0.0027	0.0026	0.0027	0.0026	0.0027	97.1
Rural track, Aggressive driver (training)	0.0068	0.0069	0.0072	0.0071	0.0070	94.1
Rural track, Offensive driver (training)	0.0027	0.0028	0.0028	0.0029	0.0028	96.9
Rural track, Normal driver (training)	0.0045	0.0046	0.0042	0.0044	0.0044	96.0
Hockenheimring track, Aggressive driver (validation)	0.0167	0.0180	0.0157	0.0158	0.0166	92.4
Hockenheimring track, Offensive driver (validation)	0.0053	0.0054	0.0046	0.0050	0.0051	96.7
Hockenheimring track, Normal driver (validation)	0.0086	0.0091	0.0070	0.0078	0.0081	95.8
Constant turn with radius of 100 m at 50 km/h (testing)	0.0013	0.0013	0.0013	0.0013	0.0013	98.9
Constant turn with radius of 100 m at 75 km/h (testing)	0.0025	0.0036	0.0044	0.0041	0.0037	97.5
Constant turn with radius of 100 m at 100 km/h (testing)	0.0393	0.0296	0.0500	0.0500	0.0431	69.4
Constant turn with radius of 30 m at 30 km/h (testing)	0.0008	0.0011	0.0011	0.0011	0.0010	98.5
Constant turn with radius of 30 m at 50 km/h (testing)	0.0148	0.0125	0.0189	0.0203	0.0169	81.0
Constant turn with radius of 60 m at 50 km/h (testing)	0.0008	0.0010	0.0012	0.0012	0.0011	99.0
Constant turn with radius of 60 m at 75 km/h (testing)	0.0313	0.0252	0.0397	0.0411	0.0349	68.4
Double lane change (ISO-3888-2) at 30 km/h (testing)	0.0014	0.0013	0.0017	0.0014	0.0015	97.6
Sine with Dwell 60 deg at 40 km/h (testing)	0.0029	0.0020	0.0031	0.0022	0.0026	97.2
Sine with Dwell 60 deg at 60 km/h (testing)	0.0101	0.0061	0.0108	0.0067	0.0087	93.3
Sine with Dwell 60 deg at 80 km/h (testing)	0.0189	0.0133	0.0203	0.0138	0.0169	90.3
Sine with Dwell 80 deg at 40 km/h (testing)	0.0037	0.0028	0.0043	0.0025	0.0034	96.3
Sine with Dwell 80 deg at 60 km/h (testing)	0.0130	0.0081	0.0145	0.0081	0.0113	91.7
Sine with Dwell 60 deg at 80 km/h (testing)	0.0284	0.0226	0.0312	0.0231	0.0266	84.8
Bumpy road at 15 km/h (testing)	0.0154	0.0164	0.0209	0.0146	0.0170	75.1
Bumpy road at 25 km/h (testing)	0.0223	0.0236	0.0298	0.0209	0.0223	77.9
Bumpy road at 32 km/h (testing)	0.0347	0.0359	0.0472	0.0390	0.0395	74.3
Slalom 18 m at 15 km/h (testing)	0.0013	0.0014	0.0016	0.0013	0.0014	96.2
Slalom 18 m at 25 km/h (testing)	0.0012	0.0013	0.0013	0.0012	0.0012	98.8
Slalom 18 m at 35 km/h (testing)	0.0025	0.0017	0.0024	0.0019	0.0021	97.5
Accuracy of all tracks combined						91.2

## Data Availability

Data are available by request from E.Š.
